# Modulated Neuroprotection in Unresponsive Wakefulness Syndrome after Severe Traumatic Brain Injury

**DOI:** 10.3390/brainsci11081044

**Published:** 2021-08-06

**Authors:** Cristina Daia, Cristian Scheau, Aura Spinu, Ioana Andone, Cristina Popescu, Corneliu Toader, Ana Maria Bumbea, Madalina Codruta Verenca, Gelu Onose

**Affiliations:** 1 Department of Medical Rehabilitation, “Carol Davila” University of Medicine and Pharmacy, 041914 Bucharest, Romania; cristinaoctavianadaia@gmail.com (C.D.); aura_ko@yahoo.com (A.S.); ioanaandone11@yahoo.com (I.A.); cristina_popescu_recuperare@yahoo.com (C.P.); corneliutoader@gmail.com (C.T.); geluonose@gmail.com (G.O.); 2 Neuromuscular Department, Clinical Emergency Hospital “Bagdasar Arseni”, 041914 Bucharest, Romania; 3 Department of Physiology, “Carol Davila” University of Medicine and Pharmacy, 050474 Bucharest, Romania; 4 Department of Neurosurgery, National Institute of Cerebro-Vascular Diseases, 041914 Bucharest, Romania; 5 Department of Medical Rehabilitation, University of Medicine and Pharmacy, 200349 Craiova, Romania; anamariabumbea@yahoo.com; 6 Neurorehabilitation Department, Clinical Neuropsychiatry Hospital, 200473 Craiova, Romania; 7 The “St. John” Emergency Hospital for Children, 800402 Galati, Romania; madverrec@gmail.com

**Keywords:** severe traumatic brain injury, unresponsive wakefulness syndrome, vegetative state, neurological assessment, neuroprotective treatment

## Abstract

Background: We aimed to assess the effects of modulated neuroprotection with intermittent administration in patients with unresponsive wakefulness syndrome (UWS) after severe traumatic brain injury (TBI). Methods: Retrospective analysis of 60 patients divided into two groups, with and without neuroprotective treatment with Actovegin, Cerebrolysin, pyritinol, L-phosphothreonine, L-glutamine, hydroxocobalamin, alpha-lipoic acid, carotene, DL-α-tocopherol, ascorbic acid, thiamine, pyridoxine, cyanocobalamin, Q 10 coenzyme, and L-carnitine alongside standard treatment. Main outcome measures: Glasgow Coma Scale (GCS) after TBI, Extended Glasgow Coma Scale (GOS E), Disability Rankin Scale (DRS), Functional Independence Measurement (FIM), and Montreal Cognitive Assessment (MOCA), all assessed at 1, 3, 6, 12, and 24 months after TBI. Results: Patients receiving neuroprotective treatment recovered more rapidly from UWS than controls (*p* = 0.007) passing through a state of minimal consciousness and gradually progressing until the final evaluation (*p* = 0.000), towards a high cognitive level MOCA = 22 ± 6 points, upper moderate disability GOS-E = 6 ± 1, DRS = 6 ± 4, and an assisted gait, FIM =101 ± 25. The improvement in cognitive and physical functioning was strongly correlated with lower UWS duration (−0.8532) and higher GCS score (0.9803). Conclusion: Modulated long-term neuroprotection may be the therapeutic key for patients to overcome UWS after severe TBI.

## 1. Introduction

The advancement of modern medicine provides a higher rate of survivors with different sequelae after traumatic brain injury (TBI), which may or may not be associated with other pathologies in the polytrauma context [1].

A common development in patients with severe TBI is the onset of coma, which is defined by the absence of arousal, sleep–wake cycles, and spontaneous eye-opening [2,3]. Currently, Unresponsive Wakefulness Syndrome (UWS) is used to describe this state [4]. UWS is a clinical syndrome describing patients who fail to show voluntary motor responsiveness in the presence of eyes-open wakefulness, which can either be transitory on the way to recovery from (minimal) consciousness or irreversible [4,5]. An older name, but still in use and very well known, for UWS is Vegetative State (VS), and Persistent Vegetative State (PVS) describes a chronic state [4,5].

According to the American Academy of Neurology, UWS criteria consist of four elements: no evidence of command following, no intelligible verbal response, no discernible verbal or gestural attempts to communicate, and no evidence of localizing or automatic motor responses [6].

Patients recovering from a coma, or UWS, transition to Minimally Conscious State (MCS), defined by the Aspen Consensus Conference by four elements: reproducible but inconsistent command following, intelligible verbalization, discernible verbal or gestural communication responses, and localizing or automatic motor responses [7].

In TBI, after the main initial trauma, secondary pathological events occur, such as brain tissue swelling, hemorrhage, and ischemia, which induce cascade cellular and metabolic processes such as excitotoxicity, free radicals-oxidative stress, metabolic dysfunction, inflammation, apoptosis-like processes, protein misfolding, calpains release, or amylogenesis, which may cause sustained damage in correlation with the genetic characteristics of the individual, therefore worsening their evolution and their prognosis after TBI [8,9].

Neuroprotection is a neurobiological type process, part of the Endogenous Defense Activity (EDA) [8]. The EDA of the nervous system is a polychronic continuous process that involves neurotrophicity, neuroprotection, neuroplasticity, and neurogenesis [10]. These fundamental biological processes lack absolute boundaries, sharing common mechanisms and overlapping [10]. The appropriate therapeutical approach is the use of multimodal medication, which also mimics neurotrophic factors and may act on a variety of secondary lesion mechanisms [11].

Taking that into account, we have chosen a sum of modulated medications for the study group, for several specific reasons:

- Actovegin, a deproteinized hemoderivative obtained by ultrafiltration from calf blood, in order to increase the cellular energy metabolism, the respiratory capacity of mitochondria, and the oxygen and the glucose uptake [12];
- Cerebrolysin, obtained by enzymatic lysis of lipid-free pig brain products, due to its pleiotropic effect acting as a neuroprotective, neurotrophic, nootropic, and neuromodulating agent, especially after TBI [8,9,10];
- pyritinolum (Encephabol), facilitates the passing of glucose across the blood–brain barrier and increases its metabolism in the neuronal tissue; it also shows antioxidant properties [13];
- L-phosphothreonine, L-glutamine, and hydroxocobalamin protect neural tissue, enhance nerve repair, and improve functional recovery after TBI [14,15,16];
- cyanocobalamin improves nerve repair and functional recovery after TBI [16];
- alpha-lipoic acid is neuroprotective by preserving blood–brain barrier permeability and by reducing brain edema probably via its anti-inflammatory and antioxidant properties in the TBI model [17];
- carotene (pro A vitamin), coenzyme Q-10, L-carnitine, DL-α-tocopherol acetate (E vitamin), and ascorbic acid are antioxidants, protecting neuronal cells from oxidative stress. DL-α-tocopherol acetate is an antioxidant with anti-inflammatory properties and also exhibits modulatory activities of autophagy [18,19,20];
- thiamine preserves mitochondrial function, preserves blood–brain barrier function, improves the glucose intake and energy status, and decreases oxidative stress, lactic acidosis, and neurogenic inflammation [21,22];
- pyridoxine has been chosen for its neuroprotection properties and behavioral function improvement in experimental studies [23].

In our study, we have performed long-term observations on patients with UWS after severe TBI who have regained consciousness and functionality after receiving a complex rehabilitation program associated with modulated neuroprotection. Additionally, in this paper, we communicate special features in these patients and describe two stages of consciousness after TBI.

## 2. Materials and Methods

We have performed a retrospective analysis on 60 patients diagnosed with UWS after severe TBI, admitted to the Neuro-Muscular Clinic Division of “Bagdasar Arseni” Hospital from 2012 until the present.

All patients provided signed informed consent, directly or through family members/legal guardians. Official approval was obtained from the “Bagdasar Arseni” Hospital Ethics Committee no. 31307/30.11.2020.

### 2.1. Inclusion Criteria

- Patients over 18 years old;
- diagnosed with severe TBI (Glasgow Coma Scale (GCS) of 3–8 points at admission) [24];
- TBI followed by coma and then UWS;
- personal history of severe TBI not older than 3 months;
- association of different comorbidities in the context of polytrauma.

### 2.2. Exclusion Criteria

- Patients with UWS induced by any pathology other than TBI;
- patients with UWS after medium (CGS of 9–12 points) or mild TBI (GCS of 13–15 points) [24] associated or not with other factors which may induce UWS;
- personal history of severe TBI older than 3 months;
- patients with any modified conscience state after severe TBI other than UWS.

### 2.3. Study Design

The 60 patients diagnosed with UWS within 3 months from a severe TBI were hospitalized for the first 6 months in the “Bagdasar Arseni” Hospital. Later on, the patients were admitted for at least one month for each evaluation. The general common evaluations were performed at 1, 3, 6, 12, and 24 months after the TBI.

The patients were divided into two groups, study and control, depending on whether the patients’ family/legal guardians offered their approval for the administration of additional neuroprotective therapies.

### 2.4. Patients’ Rehabilitation Management 

All patients received necessary and appropriate treatment for their associated conditions and comorbidities, per current good practices and according to their specific medical needs. All patients benefited from the same kinesitherapy procedures and occupational and speech therapy, all integrated into a comprehensive rehabilitation program. All patients received appropriate treatment for additional pathological conditions or complications (infection, pressure sores, dyselectrolytemia, etc.).

### 2.5. Neuroprotective Treatment

From the moment they were admitted, the patients in the study group received additional modulated neuroprotective treatment, which consisted of intermittent administration of the following medications:

- Actovegin, 400 mg/day, every day for the first year; in the second year, 400 mg/day, 21 days each month for 6 months, then 400 mg/day, 14 days each month for another 6 months;
- Cerebrolysin, starting from the second year, 10 mL/day, a set of 10 days every three months, for one year;
- pyritinol (Encephabol) 100 mg, once a day for the first 6 months, then every other two months;
- L-phosphothreonine: 20 mg/day, L-glutamine 75 mg/day, and hydroxocobalamin 500 µg/day, daily for the first two months, then 10 days per month; during the second year, 10 days every 3 months;
- alpha-lipoic acid, 600 mg/daily for the first 3 months, then daily for a month every 3 months;
- carotene 10 mg/day, DL-α-tocopherol acetate 40 mg/day, ascorbic acid 100 mg/day, for the first 3 months;
- thiamine 100 mg/day, pyridoxine 100 mg/day, and cyanocobalamin 50 µg/day, 10 days per month for the first three months;
- coenzyme Q-10, 30 mg daily for a month, every 3 months;
- L-carnitine 100 mg, daily the first month, then 10 days per month, every 3 months.

### 2.6. Evaluated Parameters

All patients were submitted to a cranial computed tomography (CT) scan, and the results were available and recorded the day after the TBI.

Based on their reliability and validity for assessing various levels of consciousness or functionality, we have chosen several scales to evaluate the patients in the two groups. 

The first instrument used was GCS, performed on day 1 after TBI as a classic instrument to classify the TBI into the following categories: severe (3–8 points), moderate (9–12 points), and mild (13–15 points) [25].

The patients were then evaluated at 1, 3, 6, 12, and 24 months after the TBI using the Extended Glasgow Outcome Scale (GOS-E), the Disability Rating Scale (DRS), the Montreal Cognitive Assessment (MOCA), and the Functional Independence Measurement (FIM).

GOS-E is an instrument developed for patients in a coma or UWS after TBI and allows for the evaluation of an initial baseline as well as ongoing measurements in order to quantify the patient’s evolution from UWS to a good recovery. Scores can range from 1 to 8, meaning (1) dead, (2) persistent vegetative state PVS, (3) lower severe disability (requires frequent help of someone to be around at home most of the time every day), (4) upper severe disability (can be left alone > 8 h during the day, but unable to travel and/or go shopping without assistance), (5) lower moderate disability (unable to work or only in a sheltered workshop), (6) upper moderate disability (reduced work capacity; resumes < 50% of the pre-injury level of social and leisure activities), (7) lower good recovery (minor problems that affect daily life; resumes > 50% of the pre-injury level of social and leisure activities), and (8) upper good recovery (no current problems related to the brain injury that affect daily life) [26].

DRS was developed for rating the cognitive and self-caring, functional, and social outcomes after severe TBI, tracing individuals from coma to community. A DRS score of 0 points represents the lack of disability, 1 point equals mild disability, 4–6 points describe a moderate disability, 7–11 points: moderate-severe disability, 12–16 points: severe disability, 17–21 points: very severe disability, 22–24 points: vegetative state, 25–29 points represent an extreme vegetative state, and 30 points are given for death [27].

MOCA is a cognitive screening instrument developed for the detection and prediction of early cognitive impairments as well as the planning of cognitive rehabilitation early in the recovery after TBI. This tool assesses multiple cognitive domains including visuospatial and executive functioning (clock-drawing task, 3 points, and a three-dimensional cube copy, 1 point), nomination (3 points), attention (6 points), language (3 points), abstraction (2 points), short-term memory (delayed recall, 5 points), orientation (6 points), and education level (1 point). A normal score is considered a minimum of 26 points out of a total 30 [28,29].

The FIM instrument is used to quantify the amount of assistance a patient requires in different situations from 1 to 7: self-care (feeding, grooming, bathing upper body dressing, lower body dressing, toileting), sphincter control, transfer, locomotion, communication, and social cognition; the score ranges from 18 (lowest) to 126 (highest) level of independence [30].

Additionally, the main cause leading to the injury as well as any other associated traumatic lesions were recorded.

### 2.7. Statistical Analysis

SPSS Statistics for Windows version 15.0 (SPSS Inc., Chicago, IL, USA) was used for statistical analysis. The distribution of patient demographic features was analyzed using descriptive statistical methods. Levene’s test for equality of variances, *t*-test for equality of means, and Z test of proportions were used for comparison of parameters. Pearson’s chi-squared test was used for comparing categorical data. Fisher’s exact test was used in analyzing 2 × 2 contingency tables. The statistical significance of the *p*-value was considered *p* < 0.05.

## 3. Results

### 3.1. Demographic Data

The study lot comprised 33 patients (15 females and 18 males) between 18 and 68 years old, with median age and standard deviation of 40 ± 14 years, standard error mean 2.383. The control lot included 27 patients (8 females and 19 males) between 24 and 89 years old, median age and standard deviation of 49 ± 19 years, standard error mean 3.811.

### 3.2. Clinical and Paraclinical Features

The overall GCS score of the patients in the study group was 4.33 ± 1.66, while the patients in the control group scored 4.48 ± 1.60, with no significant differences between the two lots (*p* = 0.7247).

The lesions identified on cerebral CT scans in all patients are shown in Table 1.

The location of the cerebral injury correlates with the outcome of the patients in terms of survival. Lesions on the brainstem were significantly deadlier than lesions located elsewhere (*p* = 0.0260). Other involved regions, such as hemispheres or basal ganglia, did not carry a statistically significant higher risk of death.

The main cause leading to the traumatic brain injury was also recorded, along with any other associated traumatic injuries (see Table 2).

In the study group only two patients died. The first patient succumbed at 670 days from the TBI, in UWS at the moment of death. The patient’s cerebral CT scan showed left temporal-parietal contusion, diffuse axonal injury (DAI), subarachnoid hemorrhage (SAH), and pons and mid brain hemorrhagic lesions. The other succumbed at 320 days from the TBI, due to a heart attack and, at the moment of death, had the following status: GOS-E = 5, DRS = 4, FIM = 110, and MOCA = 20. For this patient, the CT scan showed bilateral frontal laceration and midbrain contusion.

For the patients in the control group, the causes of death were bronchopneumonia (8 cases), cachexia (8 cases), sepsis (5 cases), sudden death (2 cases), urinary tract infection (2 cases), and gastrointestinal hemorrhage (2 cases). In the control group, all patients died on average at 122 ± 52 days after TBI, five patients being in MCS and the rest in a UWS state at the moment of death. At the time of death, five patients who had progressed to a MCS had the following mean scores: GOS-E = 2 ± 0, DRS = 24 ± 2, FIM = 29 ± 4, MOCA = 0.

### 3.3. Cognitive Evolution 

In the evaluated patients we have identified four cognitive evolution states, as follows:

1. Unresponsive Wakefulness Syndrome (UWS)

In the control group, UWS duration was 111 ± 62 days, standard error mean 19.540, GOS-E = 2 ± 0 (persistent vegetative state), DRS = 26 ± 2 (vegetative state), MOCA = 0 ± 0.

In the study group, UWS duration was 67 ± 112 days, standard error mean 11.914, GOS-E = 2 ± 0 (persistent vegetative state), DRS = 23 ± 3 (vegetative state), MOCA = 0 ± 1.

**Figure 1:**
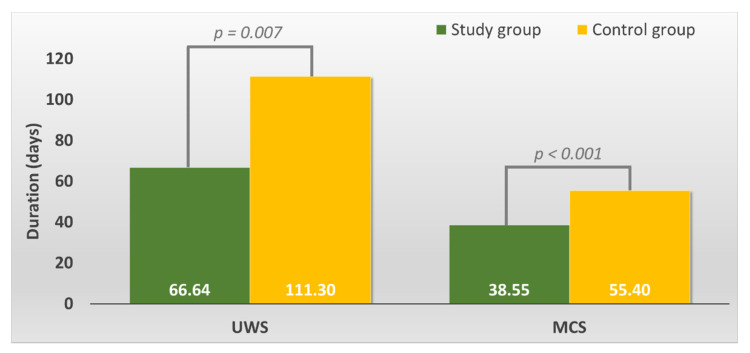
Overall duration of the Unresponsive Wakefulness Syndrome (UWS) and Minimally Conscious State (MCS) in the study versus control group.

The average UWS duration in the study group was smaller than that in the control group (*p* = 0.007, *t*-test, verify with Levene’s Test for Equality of Variances) (see Figure 1).

2. Minimally Conscious State (MCS)

In the control group, only five patients emerged to MCS at 5 ± 10 days from TBI and had a mean score of GOS-E = 2 ± 1 (vegetative state), DRS = 23 ± 1 (severe disability), MOCA = 0 ± 0. The average duration of the MCS period was 55 ± 13 days until patient death.

In the studied group, the patients emerged to MCS at 46 ± 31 days from TBI, had a mean score of GOS-E = 2 ± 1 (vegetative state), DRS = 15 ± 5 (severe disability), MOCA = 4 ± 4. The average duration of the MCS period was 39 ± 20 days.

The average MCS duration in the study group was smaller than that in the control group (*p* = 0.000, *t*-test) (see Figure 1).

3. Moderate Conscious State (MoCS)

Beyond the MCS period, the patients in the study group continued to progress, exhibiting common clinical features such as performing more complex activities, for example, the ability to speak simple statements, showing a purposeful motor response, choosing selective emotional behavior in different situations, and even partially late memory recovery. The patients needed feeding by another person, but they were able to swallow solids and liquids by themselves and were capable to ask for food; therefore, gastrostomy was no longer required. Patients had spontaneous micturition, so an indwelling catheter was not necessary. We have named this common state “Moderate Conscious State” (MoCS). In the study group, patients emerged to MoCS at 85 ± 47 days, had a mean score of GOS-E = 4 ± 1 (upper severe disability), DRS = 10 ± 5 (moderate-severe disability), MOCA = 12 ± 7. The average duration of the MoCS period was 30 ± 15 days. No patients from the control group progressed to MoCS.

4. Psycho-Cognitive state (PCS)

**Figure 2:**
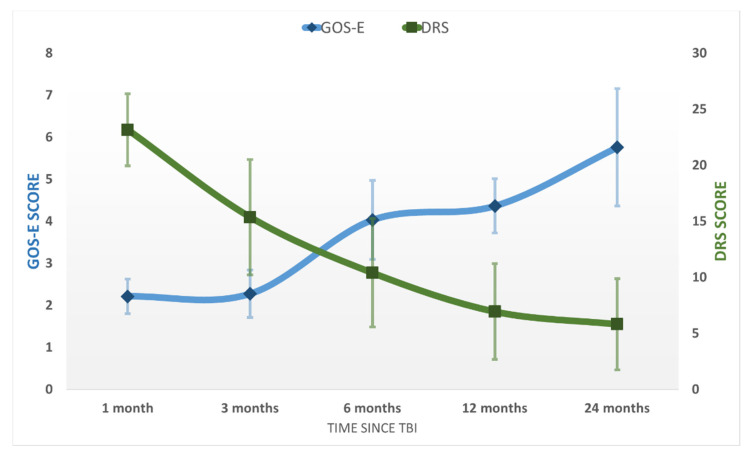
The average GOS-E and DRS scores in the study population at the five evaluations after the TBI. Error bars represent the standard deviation.

**Figure 3:**
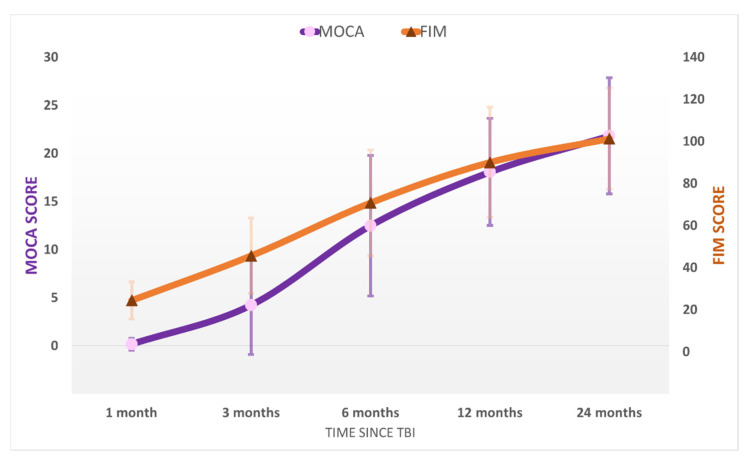
The average MOCA and FIM scores in the study population at the five evaluations after the TBI. Error bars represent the standard deviation.

After emerging from MoCS, further progress was recorded. The patients recovered more complex activities such as the ability to speak complex statements, complex behavior, and recovery of late memory but still showed problems with recent memory. The patients were able to perform purposeful motor response and locomotion and showed selective appropriate emotional behavior to a specific situation. They experienced some common symptoms such as headaches, dizziness, vertigo, fatigue, bulimia, emotional incontinence, irritability, personality disorders, depression, anxiety, restlessness, reversal of sleep–wake phases, insomnia, intellectual disorders (concentration incapacity), stress intolerance, auditive intolerance, or olfactory intolerance. We named this state the “Psycho-Cognitive state” (PCS). The patients emerged to PCS at 115 ± 59 days and showed a mean score of GOS-E = 4 ± 1 (upper severe disability), DRS = 7 ± 4 (moderate-severe disability), MOCA = 18 ± 6. At the final evaluation at 24 months, the patients of the study group had a mean score of GOS-E = 6 ± 1 (upper moderate disability), DRS = 6 ± 4 (moderate disability), MOCA = 22 ± 6 (patients reach a high cognitive level) ( Figure 2; Figure 3). No patients from the control group progressed to PCS.

### 3.4. Functional Level 

The functional level (Figure 3) of the studied patients according to their cognitive evolution was as follows:

- In the UWS period, the average FIM level was 19 ± 3 in the control group and 24 ± 9 in the study group, which functionally means the patient is bedridden in both groups.
- In the MCS state, in the control group the average FIM was 29 ± 4 (bedridden), while in the study group, FIM = 46 ± 18, functionally meaning the patient was mobilized in the specially adapted wheelchair.
- Only patients in the study group achieved the MoCS state. The average FIM was 71 ± 26 meaning, in general, that the patients maintained the sitting position in the normal wheelchair and were elevated using a tilt table for the orthostatic position.
- In the PCS the average FIM in the study group at evaluation 12 was 90 ± 27, which equivalated to patients walking with the high rolled frame assisted by the kinesiotherapist. At the final evaluation, 24 months after the TBI, the average FIM was 101 ± 25, which corresponded to assisted gait (eventually only with surveillance for those patients with higher scores).

### 3.5. Cognitive and Functional Statistical Analysis in the Study Group

In the study lot, the average of DRS increased by at least 2 points between evaluations 1 and 3 (*p* = 0.01, *t*-test), by 6 points between evaluations 3 and 6 (*p* = 0.0005, *t*-test), by 4 points between evaluations 6 and 12 (*p* = 0.008, *t*-test), and at least by 3 points between evaluations 12 and 24 (*p* = 0.006, *t*-test).

MOCA value was 0 at evaluation 1; at evaluation 3, 24.2% of patients scored higher than 7, and at evaluation 6, 69.7% of patients had values higher than 7 (*p* = 0.0001, Z test); at evaluation 12, 72.7% of patients showed scores higher than 14 on the MOCA test (*p* = 0.022, Z test); at evaluation 24, 68.7% of patients had values higher than 18 on MOCA testing (*p* = 0.029, Z test).

In the study lot, the average FIM increased by at least 17 points between evaluations 1 and 3 (*p* = 0.03, *t*-test paired), by 21 points between evaluations 3 and 6 (*p* = 0.02, *t*-test paired), by 16 points between evaluations 6 and 12 (*p* = 0.03, *t*-test paired), and by 9 points between evaluations 12 and 24 (*p* = 0.02, *t*-test paired).

**Figure 4:**
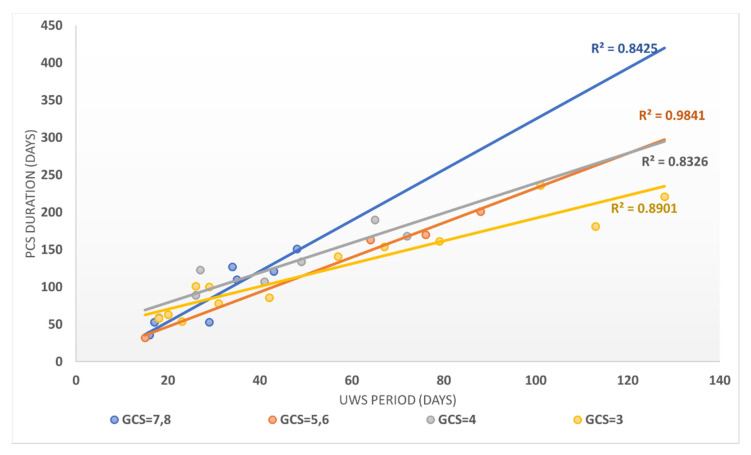
The correlation between the UWS period and the PCS duration stratified according to the GCS score for patients in the study group.

The evolution of patients in absolute terms on the FIM scale versus the UWS period (expressed in days) showed that FIM evolution strongly correlated with the UWS period (Person correlation coefficient = −0.7992). FIM evolution in relative terms was even more strongly correlated with the UPW duration (expressed in days), with a Pearson correlation coefficient of −0.8532. The duration until the transition to PCS was strongly correlated with the duration of the UWS period, as the Pearson correlation coefficient was 0.9803, stratified according to GCS in Figure 4.

## 4. Discussions

### 4.1. Limitations of the Study

The major limitation of the study is the distribution of patients, which was subjective. This was performed according to the informed consent and the decision of each patient’s family to attempt the neuroprotection treatment. Furthermore, the costs of the neuroprotection treatment were supported by the patients’ families as the hospital was unable to provide it. The patients in the control group generally did not have a supportive family to care for their needs, and the majority of these patients received social services care; furthermore, all patients in the control group died at least at 6 months after TBI, so no long-term comparisons can be performed. The support of the family for the patients in the study group may be an important additional and unmeasured factor that contributed to their positive evolution, and might even have expanded their life expectancy. For instance, the aforementioned patient from the study group survived for an extended period of time and died at 670 days from the severe TBI due to sudden death, while still in UWS.

There are few cases described in the literature that recovered from UWS [13,31]. Our study provides a larger number of cases but still is insufficient for solid conclusions. However, there are good results on long-term evaluation for the 32 patients recovering from UWS, which, according to our knowledge, is the largest number of communicated cases. Another observation of our study is the continuous progression of patients who received modulated neuroprotection. They underwent four states of consciousness: UWS, MCS, MoCS, and PCS. This point of view is original but, at the same time, restricting. We propose an original classification of the cognitive evolution after TBI; therefore, we need confirmation from similar long-term studies following patients recovering from UWS after severe TBI. Until then, the two proposed states, MoCS and PCS, should be reviewed with circumstance.

### 4.2. Neuroprotection Treatment

Following the concept that multimodal therapeutic actions could be the key to progressively improved cognitive and functional evolution of patients after TBI [10], we have chosen a series of neuroprotectors for the study group. We have attempted to target as many pathophysiological links as possible and to alternate neuroprotectors in order to increase efficiency and to allow for long-term use. In addition, the treatment was initiated early after the TBI, within 3 months, and we consider this early administration to be an important factor in the recovery from UWS.

In choosing the dosage for each medication used, we took into account the individual prescription of each drug as well as the related cited studies [8,9,10,11,12,13,14,15,16,17,18,19,20,21,22,23]. It is possible, even likely, that some of the chosen drugs, such as Cerebrolysin and Actovegin, would play a major neuroprotective role, as established by various profile studies [8,9,10,11,12]. The additional drugs used in our protocol might only carry an adjuvant potential, as we were unable to verify their individual therapeutic contribution, and this was not the aim of our study. However, the synergy of multiple drugs with various neuroprotective benefits seems to be a key approach for remobilizing the mechanisms of neural recovery and stimulating the reversal of cognitive impairment processes induced by TBI.

### 4.3. Lesions of the Brain

When considering the prognostic role of the CT evaluation, patients with lesions in the brainstem did not survive and/or did not recover from the UWS. Only patients with lesions of the cerebral hemispheres survived and recovered. The patients showed multiple focal (contusion, laceration) or diffuse (especially DAI) lesions located in various regions and associating SAH or other types of hemorrhage. The immeasurable and unsuspected possibilities of the cerebral hemispheres for functional recovery should never be underestimated [10]. However, we presume that even indirect damage to the brainstem on its dorsal side, which can occur in the secondary brain stem lesions after TBI, is a determining factor in the persistent vegetative state and ultimately in patient death [32].

### 4.4. Death of Patients in UWS after Severe TBI

Treating patients in UWS or MCS after severe TBI is challenging due to the required prevention and/or treatment of multiple systemic complications caused by immobilization (pneumonia, urinary tract infection, thromboembolic disease, pressure sores), managing multiple infections, providing good nutrition, maintaining the hydro-electrolytic balance, treating other subsequent pathology, and commencing a complex rehabilitation program, providing physical therapy to prevent limb contractures, maintain mobility, improve functionality, etc. [33]. Unfortunately, patients with UWS or MCS after severe TBI continue to die with or without neuroprotection.

### 4.5. Cognitive Evolution 

In the first conscience state, UWS, MOCA evaluation was 0 points for all studied patients. However, only the patients in the study group continued to show constant cognitive improvement and finally achieved 22 ± 6 points on MOCA testing at 24 months evaluation, with at least 68.7% scoring higher than 18 points. Taking into account the test complexity and the fact that a normal score is considered a minimum of 26 points out of a total of 30, it is important to underline that some patients in the study group recovered to a very good level of consciousness after 24 months of modulated neuroprotection.

### 4.6. Functional Level

Related to the cognitive evolution, the functional level of patients recovering from UWS reached 101 ± 25 points on FIM, meaning completely independent gait for some patients and assisted gait for others. Overall, the recovery of gait is a very important goal to achieve in this type of patient. The functional recovery rose progressively between evaluations. A shorter UWS period and a higher GCS at presentation strongly correlate with better functional evolution. It should be mentioned that the successful recovery of gait may also be sustained by the orthopedic interventions that some patients required for malpostures due to spasticity. 

### 4.7. Rehabilitation Program

A large number of physicians are involved in the recovery of a patient from UWS after severe TBI, from different specialties such as neurosurgery, neurology, intensive care medicine, rehabilitation, cardiology, infectious diseases, orthopedics, gastroenterology, thoracic surgery, plastic surgery, general surgery, and psychiatry. Our team consists of the aforementioned medical specialties, which are involved in assisting these types of patients in our hospital. In addition, the team also consists of kinesiotherapists, general nurses, physiotherapy nurses, logopedics, and ergotherapy personnel. Each team member brings specific knowledge and expertise, thus contributing to the recovery of patients with this severe pathology. Their specific contribution and support are considerable and worth mentioning. On the other hand, the modulated neuroprotection treatment helps patients play their part in the successful completion of the rehabilitation program.

## 5. Conclusions

Patients in our study who have received modulated neuroprotection treatment progressed faster and in larger numbers from UWS to higher consciousness states and also showed excellent results in recovering functional independence. We believe that the early administration of a sum of neuroprotective medications in patients with UWS after severe TBI, modulated and administered in the long-term, might be the therapeutic key to the recovery of these patients. The consciousness state of the patients recovering after UWS and later after MCS continues to improve progressively. Further studies employing a larger number of cases are required to confirm these findings.

## Figures and Tables

**Table 1:** Cerebral computed tomography findings at admission for all patients included in the study.

Lesions Identified on Brain Imaging	Study Group (*n* = 33)	Control Group (*n* = 27)
Intracerebral hemorrhage	15 (45.5%)	11 (40.7%)
Subarachnoid hemorrhage	16 (48.5%)	8 (29.6%)
Subdural hemorrhage	4 (12.1%)	2 (7.4%)
Diffuse axonal injury	8 (24.2%)	9 (33.3%)
Traumatic subdural hygroma	2 (6.1%)	2 (7.4%)
Internal capsule/thalamic contusion	4 (12.1%)	1 (3.7%)
Brainstem contusion	1 (3.0%)	5 (18.5%)
Hemispheric contusion	-	-
Frontal	18 (54.5%)	14 (51.9%)
Temporal	18 (54.5%)	12 (44.4%)
Parietal	12 (36.4)	10 (37.0%)
Occipital	1 (3.0%)	1 (3.7%)
*Bilateral*	*6 (18.1%)*	*7 (25.9%)*
Hemispheric laceration	3 (9.1%)	6 (22.2%)

**Table 2:** Overview of the leading cause of the TBI and other associated traumatic injuries sustained by the patients of our study.

Parameter	Study Group (*n* = 33)	Control Group (*n* = 27)
Cause leading to TBI	-	-
car accident	24 (72.73%)	18 (66.67%)
aggression	5 (15.15%)	5 (18.52%)
work accident	1 (3.03%)	1 (3.70%)
domestic accident	1 (3.03%)	2 (7.41%)
train accident	2 (6.06%)	1 (3.70%)
Associated traumatic injuries	-	-
thoraco-abdominal contusion	33 (100%)	27 (100%)
articular contusion (hip, knee, shoulder, elbow)	12 (36.36%)	10 (37.04%)
internal organ rupture (spleen, liver, kidney, lung)	10 (30.30%)	7 (25.93%)
calvaria or facial bones fracture	12 (36.36%)	7 (25.93%)
limbs or pelvic fracture	18 (54.55%)	15 (55.56%)
rib cage fracture	21 (63.64%)	14 (51.85%)

## Data Availability

The data presented in this study are available on reasonable request from the corresponding author.
